# High-speed nonlinear focus-induced photoresponse in amorphous silicon photodetectors for ultrasensitive 3D imaging applications

**DOI:** 10.1038/s41598-022-14330-7

**Published:** 2022-06-17

**Authors:** Andreas Bablich, Maurice Müller, Paul Kienitz, Rainer Bornemann, Charles Otieno Ogolla, Benjamin Butz, Bhaskar Choubey, Peter Haring Bolívar

**Affiliations:** 1 Institute of Graphene-Based Nanotechnology, University of Siegen, 57076 Siegen, Germany; 2 Institute of High Frequency and Quantum Electronics, University of Siegen, 57076 Siegen, Germany; 3 Micro- and Nanoanalytics Group, University of Siegen, 57076 Siegen, Germany; 4 Institute of Analogue Circuits and Image Sensors, University of Siegen, 57076 Siegen, Germany

**Keywords:** Engineering, Electrical and electronic engineering, Optics and photonics, Applied optics, Optical sensors

## Abstract

A large and growing number of applications benefit from simple, fast and highly sensitive 3D imaging sensors. The Focus-Induced Photoresponse (FIP) can achieve 3D sensing functionalities by simply evaluating the irradiance dependent nonlinear sensor response in defect-based materials. Since this advantage is intricately associated to a slow response, the electrical bandwidth of present FIP detectors is limited to a few ${\text{kHz}}$ only. The devices presented in this work enable modulation frequencies of 700 kHz and beat frequency detection up to at least 3.8 MHz, surpassing the bandwidth of reported device architectures by more than two orders of magnitude. The sensors achieve a SNR of at least $\sim 53\;{\text{dB}}$ at $115\;{\text{cm}}$ and a DC FIP detection limit of 0.6 µW/mm^2^. The mature and scalable low-temperature a-Si:H process technology allows operating the device under ambient air conditions waiving additional back-end passivation, geometrical fill factors of $100\%$ and tailoring the FIP towards adjustable 3D sensing applications.

## Introduction

3D imaging is a key technology for several frontier applications. As autonomous applications advance, the demand for fast, highly sensitive and ubiquitously integrable cameras which allow to sense distances (3D) is increasing significantly. In automobiles, 3D image sensors enable autonomous driving by continuously monitoring the scene around a car^1^ or in-cabin sensing^2^. Smart industrial applications rely on real-time 3D machine vision and machine learning that assist and facilitate sophisticated production processes^3^. A reliable and fast 3D scene analysis at low light levels in the private domain is the cornerstone for smart home, as well as for innovative virtual reality and infotainment applications^4^. In the field of life science as well as the medical sector the same requirements apply, e.g. for 3D visualization enabling non-invasive diagnosis or robot-assisted surgery^5^.

Reliable miniaturized 3D imaging systems predominantly utilize silicon-based photonic-mixer devices (PMD) or gated single photon avalanche diodes (SPADs), both exploiting the Time-of-Flight (ToF) principle that itself relies on cumbersome laser technology and sophisticated sensor architectures. Distances can be extracted either by measuring the time difference between a transmitted and a reflected light pulse or, if the light source is modulated, by using a mixing frequency^6–8^. The ToF principle requires a precise timing, hence additional extensive circuitry for accurate signal generation and processing are required. As a result, image sensor fill factors (FF) in PMD cameras are at best $\sim 22\%$^9,10^. Current SPAD based 3D camera sensors achieve even lower FF of $13\%$^11^. Alternative ToF based Light Detection and Ranging (LIDAR) distance sensors are highly sensitive but have significant drawbacks with respect to scalability, depth resolution and accuracy even in miniaturized designs^12^.

The Focused-Induced Photoresponse (FIP) is a novel and powerful 3D imaging technique where the sensor output depends on the total photon flux and on the size of the area in which they fall. In result, the areal image resolution becomes independent of the pixel size^13^. Compared to ToF based depth sensing, a read-out of the photocurrent/-voltage as well as the extensive subsequent data processing for image reconstruction is not required. FIP detectors based on the nonlinear, irradiance dependent photoresponse have already been demonstrated in various thin-film devices^13,14^ that obtain a significant density of states within the bandgap, such as organic solar cells or lead sulfide (PbS) photoconductors. As demonstrated in^13^, the read-out of two analogue sensor outputs at different focus spot positions enables:

(I) Maximum scalability (100% FF) since the depth information can directly be extracted from the detected FIP signal from a single viewpoint,
(II) Highly precise distance measurements with depth resolutions of $\pm \;0.1\%$ at $51.8 \;{\text{cm}}$ as well as long-range distance measurements up to at least $72 \;{\text{m}}$, and
(III) Low-light level detection at irradiances down to 10 µW/mm^2^ in the visible range.

For a comparison, novel high responsivity perovskite nanowires photodetectors behave nonlinearly at significantly higher irradiances $\ge 10^{4}$ µW/mm^2^ with modulation frequencies not exceeding $30\;{\text{Hz}}$^15^. High sensitivity 2D-material based photodetectors show nonlinear photocurrents at irradiances above $10^{3}$ µW/mm^2^ with response times of seconds^16,17^.

Although the FIP technique combines several advantages, its true benefit can only be achieved at high frequencies which enables the observation of fast moving objects and significantly reduces flicker noise, hence increasing the signal-to-noise ratio (SNR). Unfortunately, the cut-off frequency of state of the art sensors is presently limited to a few ${\text{kHz}}$^18^, only.

High defect densities and low charge carrier mobilities limit both, the bandwidth and responsivities in state of the art reported FIP detectors^13,14^. Physical phenomena leading to the FIP, differ significantly depending on the device architecture, the mode of operation and most importantly the material compositions and qualities.

In^13^, the FIP has been reported in highly complex dye sensitized solar cells (DSSC), whose fabrication requires additional back-end passivation to avoid moisture- and oxygen induced performance degradation. Here, trapping and de-trapping of photo-generated charge carriers in localized defect states increases the electron diffusion in a mesoporous TiO_2_ layer with increasing electron concentrations^13,19^. Depending on irradiance levels, the FIP in solar cells can be attributed either to charge carrier trapping decreasing responsivities at low intensities^20,21^ or to recombination resulting in series resistance variations when incident light intensities exceed one sun^22^. Alternatively, the FIP in post-encapsulated PbS photoconductors can be assigned to local resistivity changes^13^. PbS photoconductors are opaque prohibiting large-scale integration as single pixel sensor stacks. The organic photodetectors (OPD) proposed in^18^ also require an additional passivation and utilize a complex multilayer structure to form internal energetic barriers that screen the electric field for different irradiances causing the FIP.

In this paper, we report a high-speed detector design utilizing a well-established, low-temperature technology which significantly surpasses the bandwidth of all previously reported FIP sensor designs. The following section discusses the device technology and physics. The subsequent section presents experimental distance measurement results along with a primarily proposed sensor read-out technique based on a harmonic analysis.

## Device technology and physics

For the first time, we used an amorphous silicon *PIN* photodiode for FIP. This diode has been specifically designed to optimize the nonlinear irradiance photocurrent detection. Compared to previously reported FIP detectors, the technology and fabrication of hydrogenated amorphous silicon (a-Si:H) photodiodes is simple, mature, scalable and suitable for back end of line device integration with traditional complementary metal–oxide–semiconductor (CMOS) electronics. Beyond that, this technology enables geometrical fill factors of $100\%$^23,24^ and allows precise, application specific tailoring of the FIP. A significant advantage compared to the former architectures is that a-Si:H FIP sensors do not require an additional passivation and can easily be operated under ambient air conditions. Besides technological benefits, an additional advantage of a-Si:H towards state of the art organic and dye-sensitized devices is the long-term stability, which is a fundamental requirement for an image sensor pixel^25,26^. We first investigate an irradiance dependent current breakdown in a-Si:H photodetectors systematically by electro-optical simulations. Next, a-Si:H FIP sensors have been fabricated via plasma enhanced chemical vapor deposition (PECVD) and characterized electro-optically to validate fundamental device functionalities and reliability. Z-Scan current measurements have been performed to study the influence of the irradiance and the bias voltage on the FIP. The z-Scan technique is a well-established and widely acknowledged method to precisely quantify and characterize optical nonlinearities in solids, liquids, or solutions^27^. We adapt this technique and develop a comprehensive electro-optical simulation model of irradiance dependent current measurements on a-Si:H FIP detectors utilizing the software AFORS-HET^28^. Finally, we demonstrate AC distance measurements based on the optimized FIP sensor design for different modulation frequencies. Details about the deposition tool, process parameters, simulation procedures and the measurement setups are given in the Methods and the Supplementary Information.

### Electro-optical simulations

The simulation results enable systematic investigations on internal charge carrier statistics and transport processes and serve as input parameters for subsequent fabrication steps.

Electro-optical simulations of the complete *ITO-PIN-ITO* multilayer stack have been conducted at a wavelength of $488\;{\text{nm}}$ for different photon fluxes of ${\Phi }_{0} = 0$, ${\Phi }_{1} = 10^{14} \;{\text{cm}}^{ - 2} {\text{s}}^{ - 1}$, ${\Phi }_{2} = 10^{16} \;{\text{cm}}^{ - 2} {\text{s}}^{ - 1}$ and ${\Phi }_{3} = 10^{18} \;{\text{cm}}^{ - 2} {\text{s}}^{ - 1}$, respectively. The corresponding irradiances and detector positions in the z-Scan are given in the Supplementary Information. A bias voltage ${V}_{bias}$ of $0\;{\text{V}}$ has been used to prevent bias induced band bending and influences on the intrinsic electric field $E_{i}$.

1 $$E_{i} = \frac{{V_{bi} \pm V_{bias} }}{{d_{i} }}; eV_{bi} = E_{M} - E_{{F\left( {p - layer} \right)}} - E_{{F\left( {n - layer} \right)}}$$

Here, the built-in voltage ${V}_{bi}$ depends on the mobility gap energy ${E}_{M}$ and on ${E}_{F(n, p-layer)}$ that is the energetic distance between the Fermi levels of n-/p-type a-Si:H and the corresponding mobility edges^29^. Acceptor doping concentrations of $N_{a} = 6 \cdot 10^{19} \;{\text{cm}}^{ - 3}$ for the p-layer and donor doping concentrations of $N_{d} = 5 \cdot 10^{19} \;{\text{cm}}^{ - 3}$ for the n-type a-Si:H serve as input parameters for the simulations. These values have been calculated taking into account the atomic density of a-Si:H^30^ and appropriate literature on a-Si:H technology^29^ considering specific deposition process parameters. Bias dependent z-Scan results confirm $0\;{\text{V}}$ to be the optimal device specific operation condition (cf. Fig. S4) for enhanced 3D depth sensing applications. To the best of our knowledge, bias tunable FIP detectors have not been reported by other groups before^13,14^.

The simulations that are discussed in the following help to identify the physical origin of the FIP in a-Si:H *PIN* photodiodes. They reveal a huge variety of depth and irradiance dependent device parameters, including charge carrier generation and recombination processes and local current densities. In the following, we discuss three particular characteristics:

(I) Band profiles including the quasi Fermi energies for electrons ${E}_{fn}$ and holes ${E}_{fp}$,
(II) Charge carrier densities $\cong$ charge carriers being trapped in defect states ${Q}_{tr}$, and
(III) Electric field profiles.

**Figure 1:**
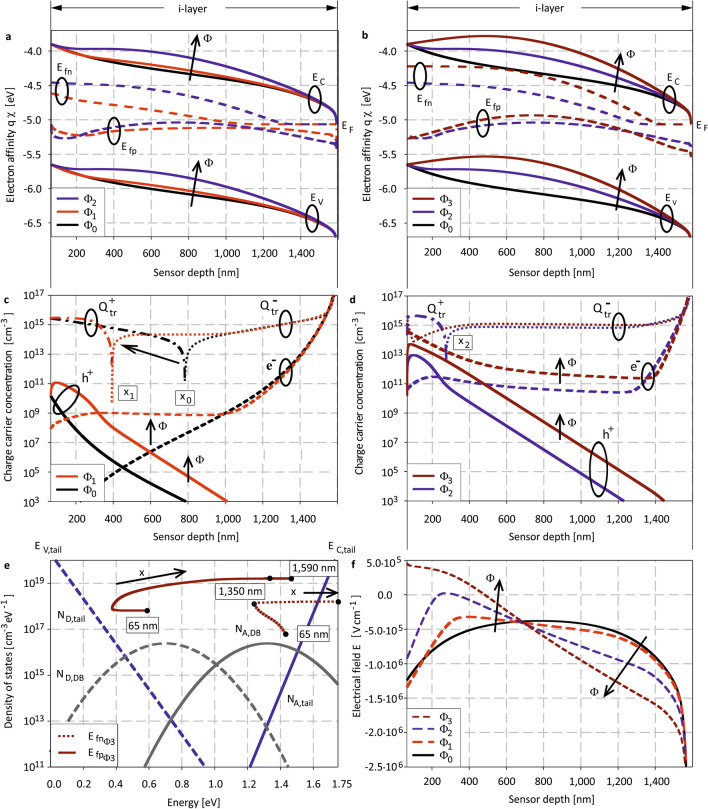
Simulated position and irradiance dependent (**a**)–(**b**) bandgap profiles including quasi Fermi level splitting under illumination, (**c**)–(**d**) charge carrier statistics, (**e**) density of state distribution including position dependent quasi-fermi levels and (**f**) electrical field distributions. At high intense illumination ${\Phi }_{3}$, ${E}_{fn}$ converges towards the conduction band tail, ${E}_{fp}$ dissociates from the valence band tail resulting in local ${e}^{-}$ (dashed lines, (**c**)-(**d**)) injection, ${h}^{+}$ (continuous line, (**c**)–(**d**)) extraction, respectively. Trapping charge ${Q}_{tr}$ that is equivalent to the total charge carrier density, becomes negative throughout the total intrinsic device region. The electrical field gets significantly distorted impeding the electron transport and resulting in a nonlinear current breakdown; the FIP.

Figure 1a–b shows simulated bandgap profiles across the intrinsic absorption layer for different irradiance levels. Under illumination, the Fermi energy ${E}_{F}$ splits into the quasi Fermi energies ${E}_{fn}$ and ${E}_{fp}$. These energy levels bend due to charge carrier injection, with ${E}_{fn}$ converging towards the conduction, ${E}_{fp}$ towards the valence band tail. Furthermore, the hole concentration ${h}^{+}$ in the device front increases moderately. However, the concentration of electrons ${e}^{-}$ significantly increases at higher irradiance levels, as shown in the charge carrier statistics (cf. Fig. 1c–d). This coincides with ${E}_{fn}$ having local convergence more intensely towards the conduction tail edge than ${E}_{fp}$ to ${E}_{V,tail}$ (cf. Fig. 1e). The position ${x}_{i}$ where the majority carrier type changes from ${h}^{+}$ to ${e}^{-}$, shifts towards the light incident side at higher irradiances (Fig. 1c–d). In the dark state ${\Phi }_{0}$, as well as at the low and moderate irradiances of ${\Phi }_{1}$ and ${\Phi }_{2}$, defect states ${Q}_{tr}$ are positively charged (solid lines) close to the p-type region, while they are negatively charged (dashed lines) close to the rear contact. Both parameters, ${x}_{i}$ and ${Q}_{tr}$ play a significant role to understand the FIP, as they determine the electrical field profile and charge carrier transport mechanism within the device. Without illumination, the majority carrier distribution of ${h}^{+}$ and ${e}^{-}$ is almost symmetric (cf. Fig. 1c) and hence the detector retains a uniform electric field across the intrinsic layer (i-layer) as predicted by Crandall considering material and device specific assumptions^31^ (cf. Fig. 1f). Furthermore, in equilibrium, electrons are able to drift to the rear contact due to the built-in field. At high intensity illumination ${\Phi }_{3}$, the ${e}^{-}$ concentration at the light incident side surmounts that of ${h}^{+}$ leading to a change in the majority carrier type. In addition, throughout the complete intrinsic region, the relationship ${E}_{C}-{E}_{fn}<{E}_{fp}-{E}_{V}$ is maintained and hence all defect states ${Q}_{tr}$ become negatively charged. This irradiance dependent defect charging influences the local electrical field (cf. Fig. 1f) and charge transport significantly, leading to a complete field collapse or even field reversal at higher fluences. Further details on internal device physics are given in^32^.

Since the electric field at the *PI*-interface of the *PIN* photodiode becomes positive, a significantly large amount of trapped and free $e^{ - }$ is not able to penetrate further, resulting in a total negative space charge $\rho$ throughout the complete absorbing region. Using Poisson’s equation

2 $$\nabla E = \frac{\rho }{{\varepsilon_{a - Si:H} }}$$

with the electric field $E$, and the relative permittivity of a-Si:H $\varepsilon_{a - Si:H}$ the negative space charge extenuates and quenches the electric field at the rear end of the device. The partial rise to potentially even positive $E$-field at the device front disturbs and—particularly in case of a field reversal—prevents charge to be collected efficiently. As a result, the total device current decreases. From the simulation results, we systematically extracted process parameters (cf. Supplementary Information) required for low temperature a-Si:H FIP detector fabrication.

## Distance measurements utilizing a-Si:H FIP sensors

### Experimental setup

The hypothesis of a significant $E$-field distortion within the FIP sensor quenching the total device current at high irradiances in the simulation coincides with the experimental z-Scan current measurement as presented in the Supplementary Information. We observe a significant current breakdown, once the a-Si:H thin-film detector is placed in focus of a 488 nm laser beam with a total power $\le 150$ µW. Compared to state of the art FIP sensors^13,14^, the FIP in a-Si:H samples occurs at irradiances down to at least $0.6$ µW/mm^2^ far out of focus (cf. Supplementary Information). This surpasses cutting-edge benchmarks by at least a factor of $16$ in the visible range. Such irradiances can easily be achieved utilizing flashlights or light emitting diodes, removing the need of expensive laser technology. Both, simulation results and experimental findings verify that the high charge carrier densities locally reduce the internal electrical field in the device, resulting in an irradiance dependent current breakdown and hence enabling via this sensitive nonlinearity optical distance measurements as elaborated in^13^.

**Figure 2:**
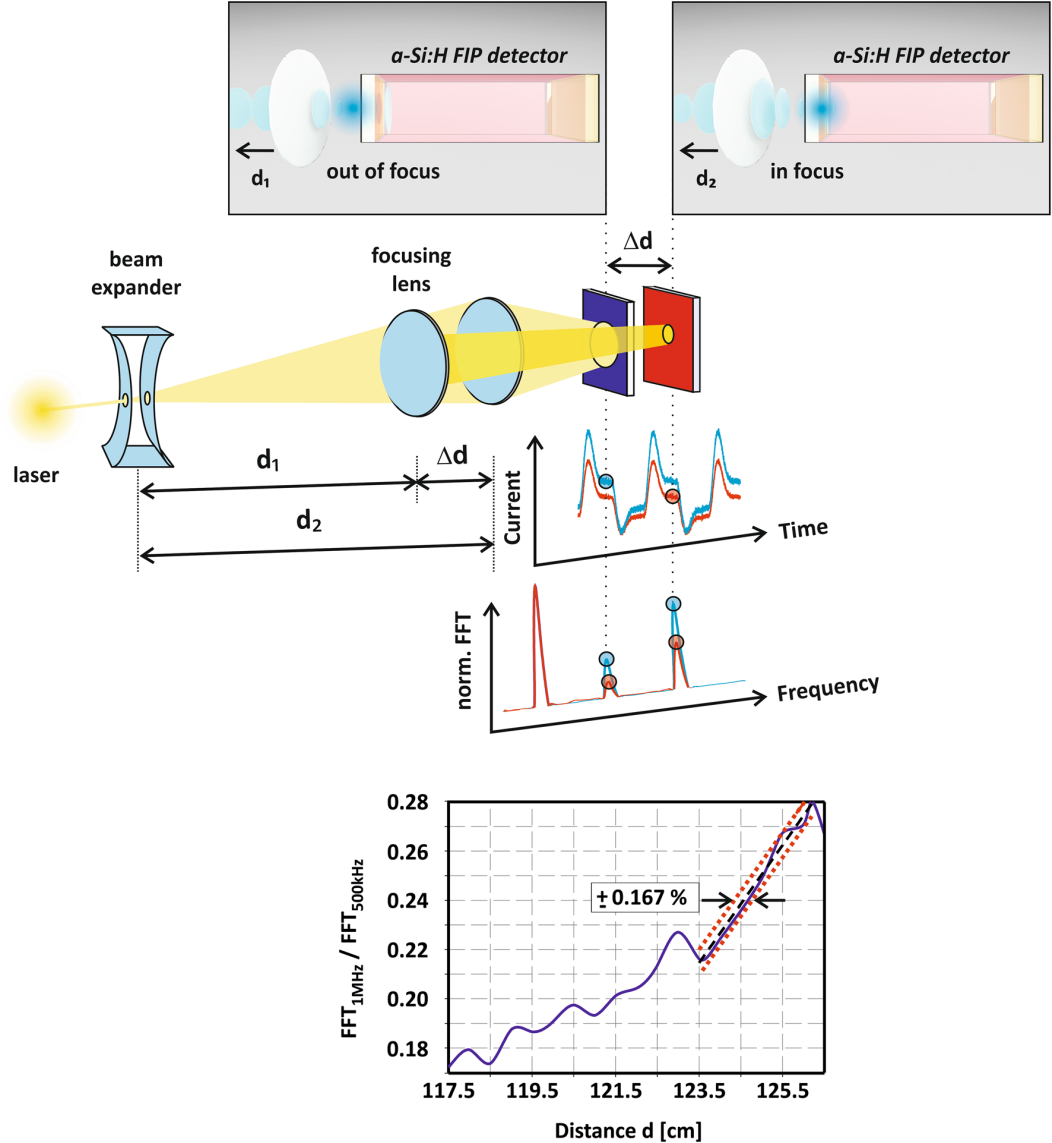
Distance measurement setup exploiting the frequency dependent FIP. The irradiance on the detector changes for different distances. Evaluating the FFT-amplitudes at specific positions in the frequency spectrum allows for unambiguous, ultrasensitive optical distance measurements utilizing a single pixel detector. The read-out approach based on a harmonics analysis enables a depth resolution of $\Delta d = 2.09\;{\text{mm}}$ at $d{ } = { }1.255\;{\text{m}}$.

The FIP in a-Si:H has been exploited and performance limits investigated for optical distance measurements at various modulation frequencies $f_{mod}$, a wavelength of $477\;{\text{nm}}$, $0\;{\text{V}}$ bias and distances exceeding $1\;{\text{m}}$. A schematic of the setup for optical measurements is shown in Fig. 2. Initially, the single-pixel FIP sensor has been placed in focus at a specific distance $d$. The diverging lens converts a collimated laser beam into a radial emitter. The converging lens then focuses the collected photons on the detector. Compared to previous reports, we propose an alternative FIP sensor readout to extract ultrasensitive distance information instantaneously from the incident light by applying a Fast Fourier Transform (FFT) on the sensor output at different distances. This novel approach based on a harmonic analysis allows for single-pixel distance determination, whereas previous concepts invariably relied on two separate FIP sensors and a signal comparison of two detector outputs^13,14,18^. The proposed sensor readout in this work can easily be integrated on-chip, e.g. by embedding two narrow bandpass filters for signal acquisition at two specific measurement frequencies and a current/voltage divider circuit to determine signal quotients for unambiguous distance determination. Hereby, the low-temperature a-Si:H deposition technique allows for sensor integration on top of the read-out circuitry providing geometrical pixel fill factors of 100%^23,24^. Further experimental details are given in the Methods section.

### Time domain versus harmonic analysis

**Figure 3:**
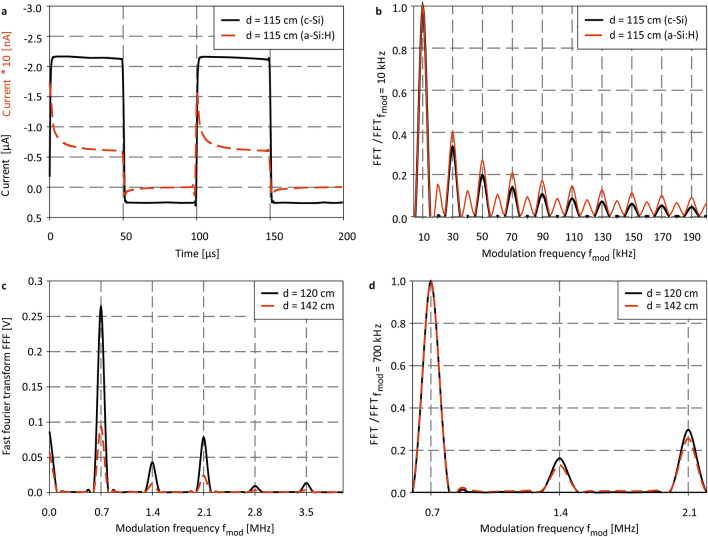
(**a**) Time-domain and (**b**) normalized absolute FFT amplitudes of c-Si (black) and a-Si:H (red) at $d = 115\;{\text{cm}}$, $477\;{\text{nm}}$ and $0\;{\text{V}}\;{\text{bias}}$. In (**b**), the signals have been normalized on the peak amplitude at ${f}_{mod}$. Independent from irradiance and distance, normalized absolute FFT amplitudes at the harmonic frequencies $\left( {2 \cdot {\text{n}} + 1} \right) \cdot f_{{mod}}$ remain constant for the c-Si detector. Nonlinear beat frequencies only occur in the a-Si:H FIP detector due to signal overshoots at the rising and falling edges of the transient detector response. (**c**) Absolute FFT signals recorded at $d = 120\;{\text{cm}}$ and $d = 143\;{\text{cm}}$ for $f_{mod} = 700\;{\text{kHz}}$, $0\;{\text{V}}\;{\text{bias}}$ and $477\;{\text{nm}}$, and (**d**) absolute FFT signals normalized on $f_{mod} = 700\;{\text{kHz}}$ verifying an amplitude distinction at $2.1\;{\text{MHz}}$, enabling unambiguous distance determination.

The time domain detector current and the corresponding rectangular modulation at $f_{mod} = 10\;{\text{kHz}}$ and $d = 115\;{\text{cm}}$ are shown in Fig. 3a. The normalized absolute FFT signals are given in Fig. 3b. While the rectangular shape of the modulation is clearly visible at the sensor output, further overshoots occur at the rising and falling signal edges as a result of photo-induced storage charges in illuminated a-Si:H *PIN* photodiodes^33^. These signal overshoots in the time domain can be explained by a slow filling and discharging of trapping states, predominantly of deep dangling bond states^34,35^. Although frequency domain a-Si:H photodiode signals already have been studied extensively in the past, anomalies besides the typical harmonic frequencies have not been reported, yet^33^. Conventional low-defect monocrystalline silicon (c-Si) photodetectors (S1337-33BQ, Hamamatsu) neither show significant capacitive (de-)charging in the time domain, nor a beat frequency generation in the frequency domain (cf. Fig. 3b). In order to maximize the sensor sensitivity, we conducted frequency dependent distance measurements as the $1/f$ noise in a-Si:H *PIN* photodiodes can be reduced significantly at higher frequencies^36^. For this, it is important to determine the RC time-constant of the photodetector since the sensor itself acts as a low-pass filter quenching its output at frequencies extending $1/RC$. At $0\;{\text{V}}$ bias, the a-Si:H FIP detector we used for optical distance measurements obtains a series capacitance of $C = 177,17\;{\text{pF}}$ and a series resistance of $R = 12,3\;{\Omega }$. The resulting time constant of $\tau_{a - Si FIP} = 2.18$ µs corresponds to a cut-off frequency of $f_{c} \approx 459\;{\text{kHz}}$, increasing $f_{c}$ of the fastest reported non a-Si:H FIP detector by a factor of $23$. As a result, we studied the FIP around the center cut-off at different modulation frequencies of $f_{mod} = 200\;{\text{kHz}}$ and $f_{mod} = 700\;{\text{kHz}}$. Figure 3c–d show FFT signals for two distances at $d = 120\;{\text{cm}}$ and $d = 142\;{\text{cm}}$ for $f_{mod} = 700\;{\text{kHz}}$, surpassing modulation frequencies of cutting-edge FIP detectors exactly by a factor of 35^14^. At the position $d = 142\;{\text{cm}}$, the sensor is placed in focus since the FFT signals increase for shorter distances due to the FIP. Besides the expected harmonics at the frequency positions $\widehat{\upxi }=\left(2\cdot n+1\right)\cdot {f}_{mod}$, additional beat frequencies occur in the frequency spectrum at $\widehat{\Psi }=\left(2\cdot n\right)\cdot {f}_{mod}$. The normalized FFT amplitudes are given in Fig. 3d showing the frequency spectrum covering $600\;{\text{kHz}}$ up to $2.2\;{\text{MHz}}$. Here, the nonlinear current breakdown can be verified clearly at $2.1\;{\text{MHz}}$ enabling unambiguous distance determination up to that frequency range by comparing signal amplitudes at the frequency positions $\widehat{\Psi }$ and $\widehat{\upxi }$.

**Figure 4:**
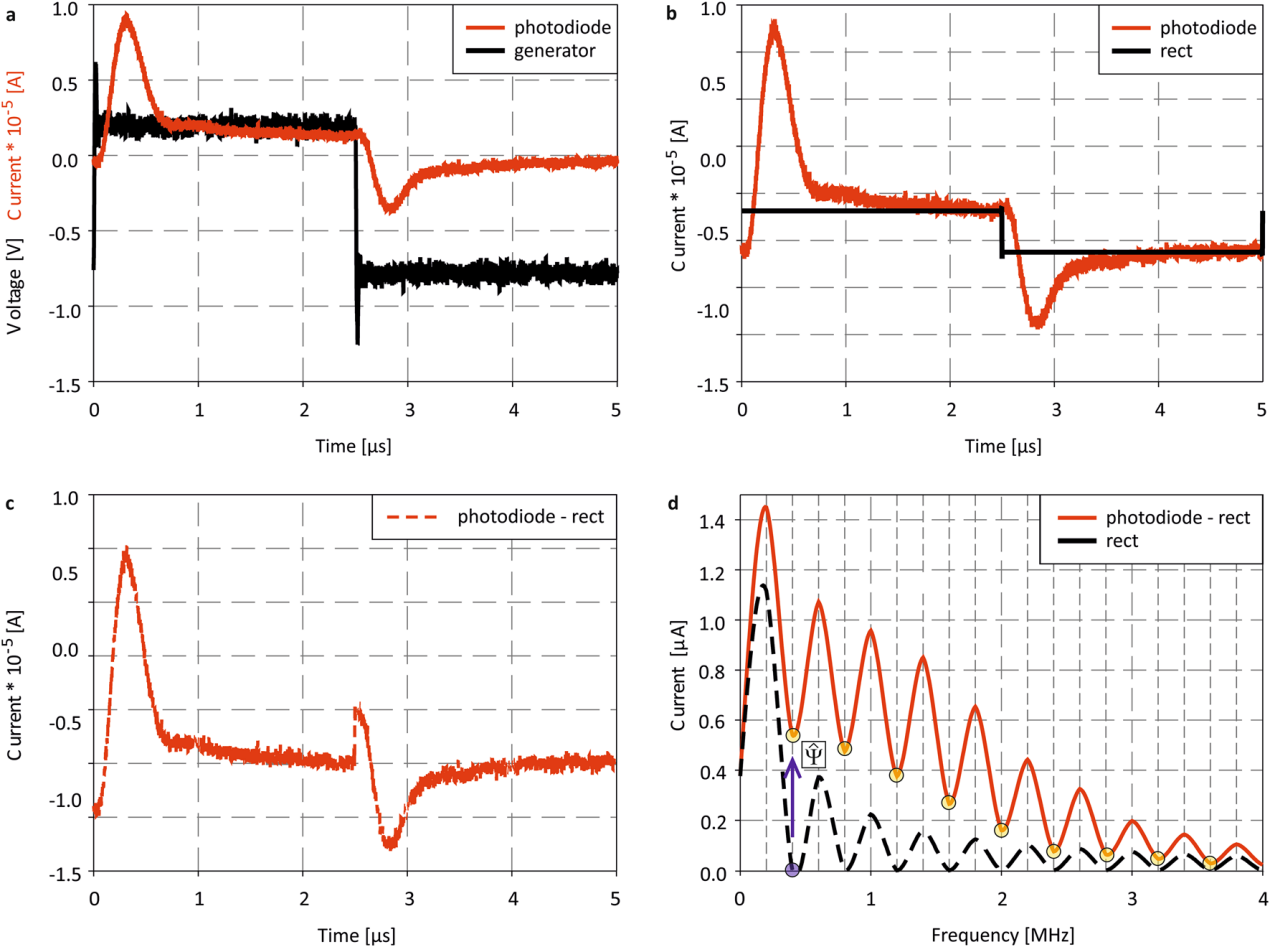
(**a**) Time domain signal of the a-Si:H FIP detector output (red) and rectangular modulation (black) at $0\;{\text{V}}\;{\text{bias}}$, $477\;{\text{nm}}$ and $f_{mod} = 200\;{\text{kHz}}$. (**b**) Time domain signal of the a-Si:H FIP detector output (red) and the fitted, optimized sensor output (black). (**c**) Difference between the time domain signal with the fitted rectangular and (**d**) FFT of the signal shown in (**c**) (red line) and the rectangular output (black).

We further analyzed the time domain sensor signal at $f_{mod} = 200\;{\text{kHz}}$ to study the beat frequencies origin, their evolution, and, most importantly the sensor sensitivity at a specific distance and frequency below ${f}_{c}$ (cf. Fig. 4a). In the first step, the defect-induced overshoot has been separated from the idealized rectangular detector output that has been fitted considering the steady-state current values (cf. Fig. 4b). The difference between the detector current and the fitted rectangular in the time domain is shown in Fig. 4c. The corresponding frequency domain signals are given in Fig. 4d. While the a-Si:H detector output obtains significant beat frequencies at the positions ${f}_{beat}=\left(2\cdot n\right)\cdot {f}_{mod}$, the FFT amplitude of the rectangular signal vanishes by definition at these spot frequencies (cf. Fig. 4d). These measurement results unambiguously verify that the defect-induced signal overshoots in the time domain can be identified to be the origin of additional beat frequencies. This can easily be exploited for enhanced, high-sensitivity optical distance measurements by measuring peak amplitudes at $\widehat{\Psi }$ or $\widehat{\upxi }$ relative to ${f}_{mod}$ for various distances. In order to achieve high sensitive distance measurement, it is important to evaluate the optimum modulation (${f}_{mod}$) and measurement (${f}_{meas}$) frequency to maximize the SNR, hence the sensors sensitivity.

### Signal-to-noise

**Figure 5:**
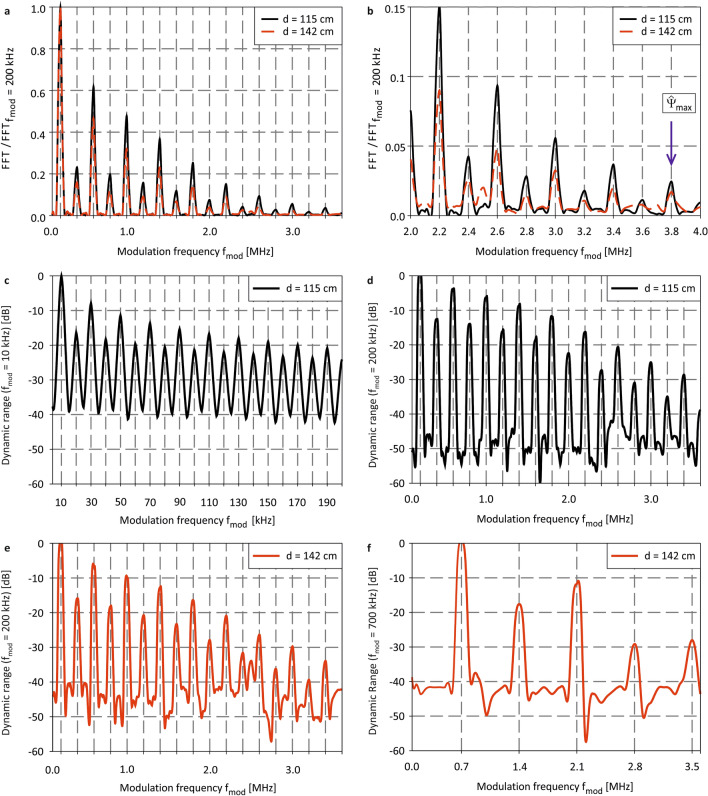
(**a**) FFT normalized on the peak amplitude of *f*_*mod*_ = $200\;{\text{kHz}}$ for $d_{1} = 115\;{\text{cm}}$ and $d_{2} = 142\;{\text{cm}}$ at $0\;{\text{V}}\;{\text{bias}}$ and $477\;{\text{nm}}$ and (**b**) close-up for a frequency spectrum from $2\;{\text{to}}\;4\;{\text{MHz}}$. The close-up reveals that a distinction of peak amplitudes at $3.8\;{\text{MHz}}$ is possible allowing for unambiguous distance determination. (**c**) SNR for $f_{mod} = 10\;{\text{kHz}}$ and (**d**) $f_{mod} = 200\;{\text{kHz}}$ at $d = 115\;{\text{cm}}$. (**e**) SNR for $f_{mod} = 200\;{\text{kHz}}$ and (**f**) $f_{mod} = 700\;{\text{kHz}}$ at $d = 142\;{\text{cm}}$. Throughout the complete spectrum, a higher SNR could be achieved for $200\;{\text{kHz}}$.

Figure 5a shows the normalized absolute FFT signals for a rectangular modulation of $f_{mod} = 200\;{\text{kHz}}$ at $d = 115\;{\text{cm}}$ and $d = 142\;{\text{cm}}$ and $0\;{\text{V}}$ bias. At $142\;{\text{cm}}$, the sensor is placed in focus. A reliable distance distinction up to at least ${\hat{\xi }} = 3.8\;{\text{MHz}}$ for $f_{mod} = 200\;{\text{kHz}}$ is possible (cf. Fig. 5b). This surpasses existing benchmarks of complex dye-sensitized solar cell FIP sensors far more than three orders of magnitude in the visible range^13,14,18^. Measuring peak amplitudes at two different frequency positions $n \cdot f_{mod} \wedge m \cdot f_{mod} ; n \ne m; n,m \ne 0$ not only allows for a fast distance determination and distinction, but also increases the SNR at higher measurement frequencies since the $1/f$ noise in a-Si:H *PIN* photodiodes can be reduced significantly at higher frequencies^36^. A significant SNR improvement from $\sim 15\;{\text{dB}}$ to $\sim 53\;{\text{dB}}$ at $200\;{\text{kHz}}$ reveals Fig. 5c–f. Since the FIP detector response is $RC$-limited, the SNR decreases at its maximum modulation of $f_{mod} = 700\;{\text{kHz}}$ compared to $f_{mod} = 200\;{\text{kHz}}$. The high SNR values throughout the complete frequency spectrum for $200\;{\text{kHz}}$ modulation consistently indicate that the irradiances can be further decreased significantly in futures distance measurement experiments.

### Depth resolution

To further estimate the achievable depth resolution $\Delta d$ of this device specific single sensor readout approach and to eliminate influences of the total light power as shown in Fig. 2, we further define a FIP detector current ratio for two different measurement frequencies

3 $$\frac{{I_{1} \left( { f_{{{\hat{\Psi }}_{1} , \hat{\xi }_{1} }} } \right)}}{{I_{2} \left( {f_{mod} , f_{{{\hat{\Psi }}_{2} ,\hat{\xi }_{2} }} } \right)}}$$

In Fig. 2 (bottom), the distance dependent evolution of this ratio is exemplarily shown for the 1st nonlinear beat frequency $f_{1} = f_{{{\hat{\Psi }}_{1} }} = 1\;{\text{MHz}}$ and a modulation nearby the sensors cut-off frequency $f_{2} = f_{mod} = 500\;{\text{kHz}}$. The maximum relative deviation of this ratio corresponds to the achievable depth resolution at a specific distance. Fitting the signal curvatures slope with a linear regression results in $\pm \;0.167\%$ precision at a distance of $d = 1.255\;{\text{m}}$, that corresponds to a depth resolution of $\Delta d = 2.09\;{\text{mm}}$. Achieving $\pm \;0.167 \%$ precision coincides with results previously reported in^13^, but at far more than twice the distance verifying significant performance improvements and potentials of this highly sensitive amorphous silicon based sensor approach and read-out concept. We believe further improvements on the bias and irradiance dependent current breakdown in a-Si:H photodiodes can be achieved in the future by optimizing:

(I) Intrinsic sensor parameters (thin-film architecture and composition),
(II) Extrinsic operation parameters (sensor bias, wavelength and modulation), and
(III) Optics (focal length, numerical aperture).

A performance comparison between state of the art FIP detectors and the results achieved and reported in this work are given in Table 1.

**Table 1:** FIP detector parameters and performance comparison including: material composition and detector type, illumination wavelength, maximum modulation *f*_*mod*_ and measurement frequency *f*_*meas*_*.*_*max*_, number of required thin-film layers and encapsulation, bias tunability, and number of pixel required for 3D measurements.

Material/device	λ [nm]	${f}_{mod}$ [Hz]	${f}_{meas, max}$ [Hz]	Layer no /Encapsulation	Bias Tunable	Sensor Count	Refs.
TiO_2_ based DSSC	530	965	965	6/yes	No	2	^13^
PbS/photo-conductor	1.550	606	606	N/A/yes	No	2	^13^
TiO_2_ based DSSC	730 /850	–	1.000	5/yes	No	2	^14^
BDP-OMe:C60 based OPD	850	20.000	20.000	5/yes	No	2	^18^
a-Si:H/*PIN* photodiode	477	**700.000**	**3.800.000**	5/**no**	**Yes**	**Single** **Pixel**	**This** **work**

## Conclusion

The bias and irradiance dependent current breakdown in a-Si:H *PIN* photodiodes has been investigated systematically by electro-optical simulations and utilized for optical distance measurements. The FIP can physically be explained by electric field screening within the intrinsic layer due to defect-assisted charge carrier trapping. We identify a local and irradiance dependent reversal of the electrical field due to localized occupied trapping states. The field reversal quenches the drift region for electrons drastically and limits the charge carrier transport at higher intensities. The a-Si:H FIP sensor and the primarily proposed read-out based on harmonic analyses enable measuring distances at modulation frequencies up to of $700\;{\text{kHz}}$, beat frequency detection up to at least $3.8\;{\text{MHz}}$ at a distance of at least $1.42\;{\text{m}}$, and depth resolutions down to $2.09\;{\text{mm}}$. At $200\;{\text{kHz}}$, we achieve a maximum SNR of $\sim { }53\;{\text{dB}}$. At continuous wave illumination, the a-Si:H FIP sensor exhibits a detection limit of at least $380\;{\text{nW}}$, corresponding to an irradiance of $0.6$ µW/mm^2^. Utilizing the flexible, low-temperature and mature PECVD technology, sensor architectures and material compositions can further be developed towards fast, highly sensitive long-range distance measurements. Since the a-Si:H device fabrication is reproducible, scalable and allows for sensor integration on top of silicon electronics with fill factors of $100\%$, this approach enables significant performance improvements of 3D imaging systems compared to existing technologies.

## Methods

### Device fabrication

**Figure 6:**
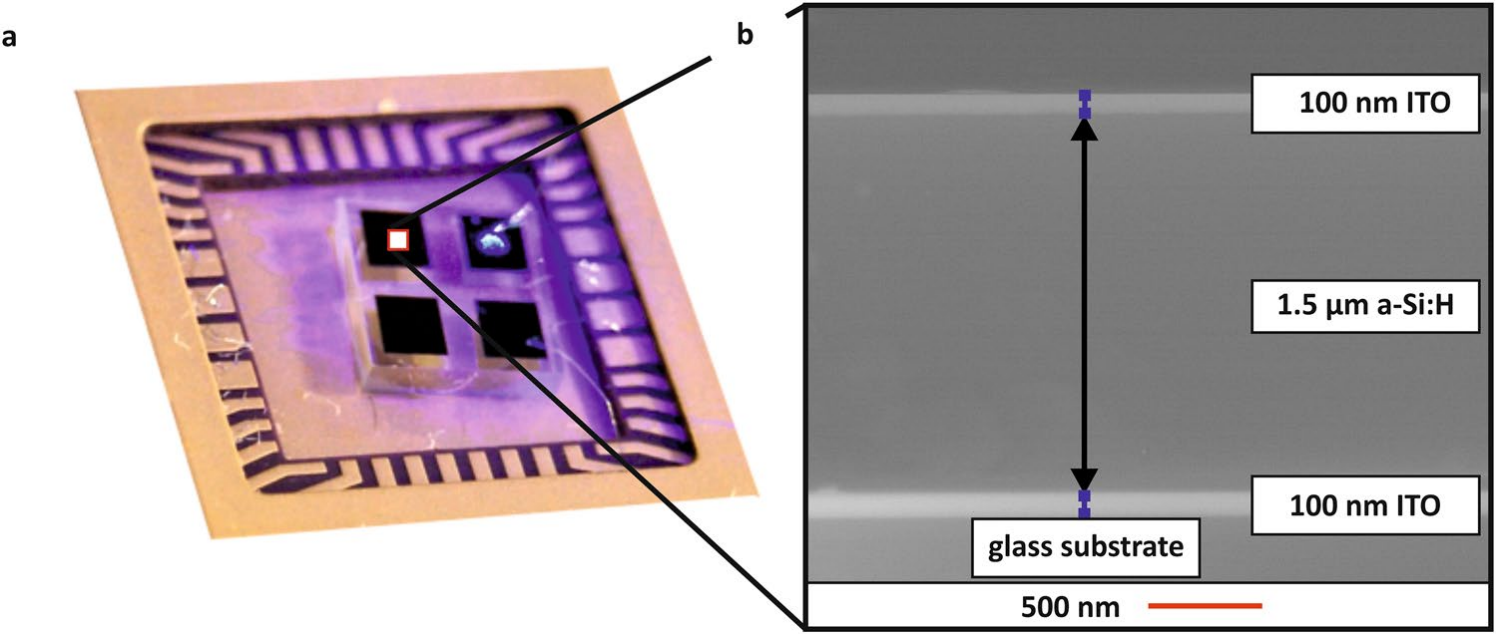
(**a**) Photograph, and (**b**) geometrically corrected cross-sectional ESEM micrograph (owing FIB cross-sectioning) of an a-Si:H *PIN* FIP photodetector.

A-Si:H *PIN* photodiodes were grown onto glass substrates in a conventional plasma-enhanced CVD (PE-CVD) process in a MVS multi-chamber vacuum system at substrate temperatures below $300\;^\circ {\text{C}}$. Anode and cathode contacts made of indium tin oxide (ITO) were deposited in a hot-wall radio-frequency sputtering system at temperatures below $50\;^\circ {\text{C}}$. Subsequent to these depositions, all devices have been thoroughly cleaned, structured by contact UV-lithography, packaged and contacted via semi-automated wedge bonding. The Supplementary Information provides further fabrication details. To characterize thin film layer thicknesses, deposition homogeneity and reproducibility, measurements utilizing a FEI Quanta 250 environmental scanning electron microscope (ESEM) have been performed on the devices in a nitrogen atmosphere. An ESEM measurement of the TCO- *PIN* -TCO multilayer stack is exemplarily shown in Fig. 6. Prior to these analyses, cross-sections of the devices have been prepared with a FEI Helios NanoLab600 focused ion-beam (FIB). The total device thicknesses have also been validated by a Bruker Dektak XT profilometer.

### Simulations

Electro-optical simulations of the z-Scan current measurement utilize a Gaussian beam profile to determine the spot size and the total photon flux on the detector serving as input parameters for the AFORS-HET simulation software^28^. To calculate the total sensor current, the simulation model takes the illuminated and dark detector areas into account. Further details are given in the Supplementary Information.

### Distance measurements

Modulated distance measurements have been performed using an Omicron LDM473.20A350 laser with a peak wavelength of $477\;{\text{nm}}$. The power on the detector has been determined by a crystalline silicon reference detector. Utilizing the technique described in the Supplementary Information of^13^, the optical power on the sensor of $7.6\;{\text{mW}}$ corresponds to an irradiance of $\sim { }2 \cdot 10^{18} \;{\text{cm}}^{ - 2} {\text{s}}^{ - 1}$. The incident photons have been guided through a diverging lens with a focal length of $30\;{\text{mm}}$ to generate a light cone similar to that of a homogenous emitting object. A lens with a focal length of $160\;{\text{mm}}$ has been used to focus the beam on the sensor, which has been positioned at variable distances behind the lens. The detector current has been converted to a voltage and amplified using a FEMTO DHPCA 100 I-V converter. Transient and FFT signal acquisition has been realized with a Tektronix TDS 3034C digital oscilloscope exhibiting a bandwidth of $300\;{\text{MHz}}$ and $2.5\;{\text{GS}}/{\text{s}}$. The signals have been recorded as an envelope of 512 measurements. Depth resolution measurements utilize a $444\;{\text{nm}}$ laser light source with an optical power on the sensor of $4.1\;{\text{mW}}$.

## Supplementary Information

Supplementary Information.

## Data Availability

Data available on request from the corresponding author.
