# Supplementary material for: High-speed nonlinear focus-induced photoresponse in amorphous silicon photodetectors for ultrasensitive 3D imaging applications
Source: Sci Rep. 2022 Jun 17;12:10178. doi: 10.1038/s41598-022-14330-7 (PMC9205940; doi:10.1038/s41598-022-14330-7)
Supplement: Supplementary file 1 — Supplementary Information. [file 41598_2022_14330_MOESM1_ESM.docx]

**Supplementary Information**

**High-Speed Nonlinear Focus-Induced Photoresponse in Amorphous Silicon Photodetectors for Ultrasensitive 3D Imaging Applications**

Andreas Bablich^1,*^, Maurice Müller^2^, Paul Kienitz^1^, Rainer Bornemann^2^, Charles Ogolla Otieno^3^, Benjamin Butz^3^, Bhaskar Choubey^4^ and Peter Haring Bolívar^1,2^

^1^ Institute of Graphene-based Nanotechnology, University of Siegen, 57076 Siegen, Germany

^2^ Institute of High Frequency and Quantum Electronics, University of Siegen, 57076 Siegen, Germany

^3^ Micro- and Nanoanalytics Group, University of Siegen, 57076 Siegen, Germany

^4^ Institute of Analogue Circuits and Image Sensors, University of Siegen, 57076 Siegen, Germany

* corresponding author

[**Fabrication parameters** 1](#_Toc95247098)

[**Electro-optical characterization (j-V, SR)** 2](#_Toc95247099)

[**Experimental z-Scan results** 3](#_Toc95247100)

[**z-Scan simulation irradiances Φ** 5](#_Toc95247101)

## **Fabrication parameters**

5 cm x 5 cm glass with a high transition temperature (Schott AF32 Eco) serve as substrates for the multilayer thin-film stacks. Prior to the deposition processes, all samples have been thoroughly cleaned: I.) in Aceton for 3 minutes in an ultrasonicaton bath (cleaning DI-water and N_2_ dry) and II.) in Isopropanol bath for 3 minutes in an ultrasonicaton (cleaning DI-water and N_2_ dry). Subsequent layers have been deposited in a high vacuum MVS multi-chamber cluster-tool including four PE-CVD deposition chambers operating at radio-frequency of 13.56 MHz (cf. Figure S1).


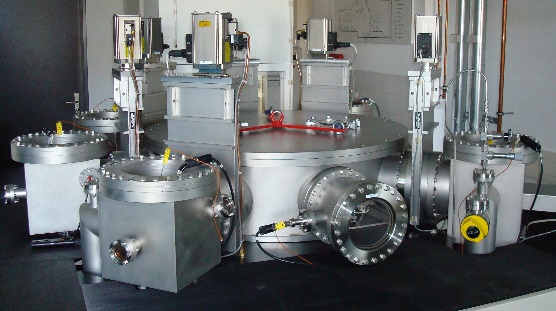


Figure S1: PECVD multi-chamber system

A separate PVD sputtering system at 13.56 MHz has been used for indium tin oxide (ITO) contact deposition. Silane (SiH_4_) serves as a precursor for a-Si:H. Additional phosphine (PH_3_) and diborane (B_2_H_6_) has been added to realize n-/p-type a-Si:H. Detailed process parameters are given in Table S 1. The devices have been structured to 1.6 mm x 1.8 mm by contact UV-lithography. ITO has been etched using hydrochloric acid (HCl) with a concentration of 5.5 %. Dry-etched of a-Si:H has been realized in a reactive ion etching system (SWAFER, Cobrain) with 100 sccm SF_6_, 50 sccm Ar at 100 W and 0.133 mbar.

| Detector type | a-Si:H pin photodiode |
| --- | --- |
| Cathode | ITO |
| RF-power [W] / time [s] | 100 / 120 |
| Pressure [mbar] | 0.055 |
| chamber temperature [C°] | 40 |
| n-a-Si:H |  |
| SiH_4_ [sccm] / PH_3_ [sccm] | 20 / 15 |
| RF-power [W] / time [s] | 6.9 / 120 |
| Pressure [mbar] | 0.667 |
| chamber temperature [C°] | 200 |
| i-a-Si:H |  |
| SiH_4_ [sccm] | 20 |
| RF-power [W] / time [s] | 7.1 / 3000 |
| Pressure [mbar] | 0.667 |
| chamber temperature [C°] | 200 |
| p-a-Si:H |  |
| SiH_4_ [sccm] / B_2_H_6_ [sccm] | 20 / 15 |
| RF-power [W] / time [s] | 6.9 / 120 |
| Pressure [mbar] | 0.667 |
| chamber temperature [C°] | 200 |
| Anode | ITO |
| RF-power [W] / time [s] | 100 / 120 |
| Pressure [mbar] | 0.055 |
| chamber temperature [C°] | 40 |

Table S 1: Deposition parameters of the a-Si:H FIP detector.

## **Electro-optical characterization (j-V, SR)**

Current density-voltage measurements (j-V) with and without standardized AM1.5 illumination have been performed using a Keithley 4200-SCS parameter analyzer and a Suss microprobe station to verify the functionality and reproducible performance of the fabricated photodetectors. J-V characteristics under illumination and dark conditions are shown in Figure S3a. For bias dependent spectrally resolved photoresponse measurements (SR), a monochromator (Acton Research, tungsten-halogen lamp) has been used to sample narrow wavelength bands of 10 nm in the range of 300 nm to 700 nm. The photocurrent signals have been converted in a voltage using a current amplifier (DLCPA-200, FEMTO Messtechnik GmbH, Germany). The data have been processed utilizing lock-in technique (Princeton 5210). The optical setup was calibrated with a reference c‑Si photodetector (S1337-33BQ, Hamamatsu) to extract absolute spectral responsivity values. The bias dependent spectral response of the a-Si:H FIP detector is shown in Figure S3b. The general behavior corresponds to the well-known behavior of a‑Si:H pin-diodes. All electrical and optical measurements have been conducted under ambient conditions. To proof device reliability and reproducibility, j-V and SR measurements have been performed at least 3 times on more than 10 different device samples.


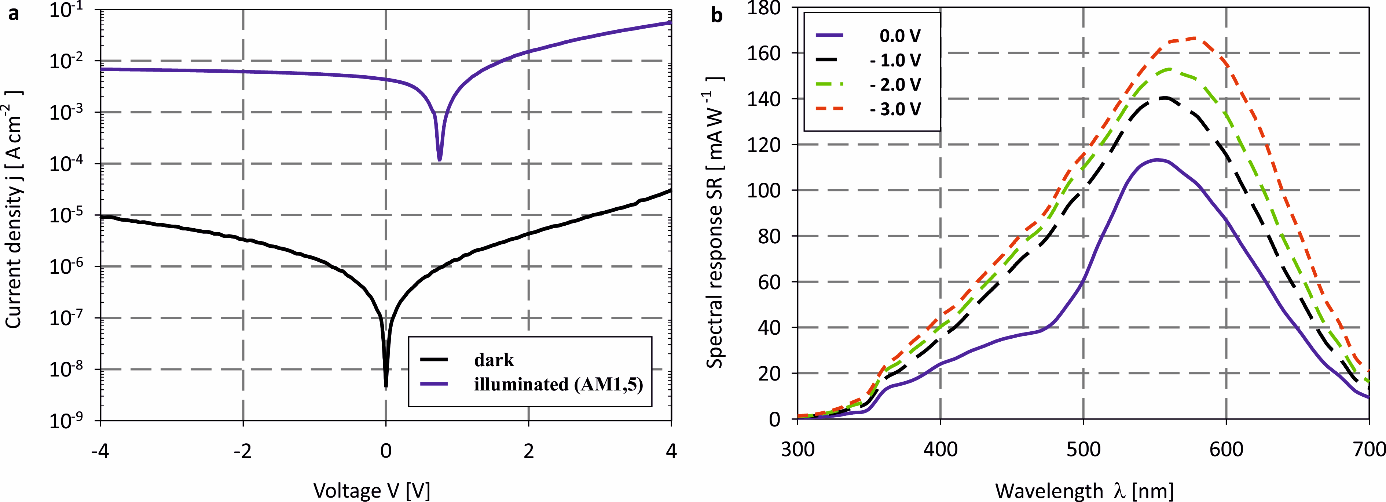


Figure S2: Electro-optical characterization of the a-Si:H pin FIP detector: (a) j-V characteristics and (b) voltage dependent spectral response measurement.

## **Experimental z-Scan results**

*Figure S3* shows the experimental z-Scan measurement results at different light intensities for 488 nm. Due to the FIP, absolute sensor currents do scale nonlinear with the incident light power and are therefore plotted in logarithmic scale. The dynamic of the current breakdown is slightly reduced at higher overall optical power values. Compared to previous FIP sensors and architectures, the FIP in a‑Si:H *PIN* photodiodes occurs at irradiances down to at least $0.6 \mu W/mm^{2}$ far out of focus (z = 600 µm).


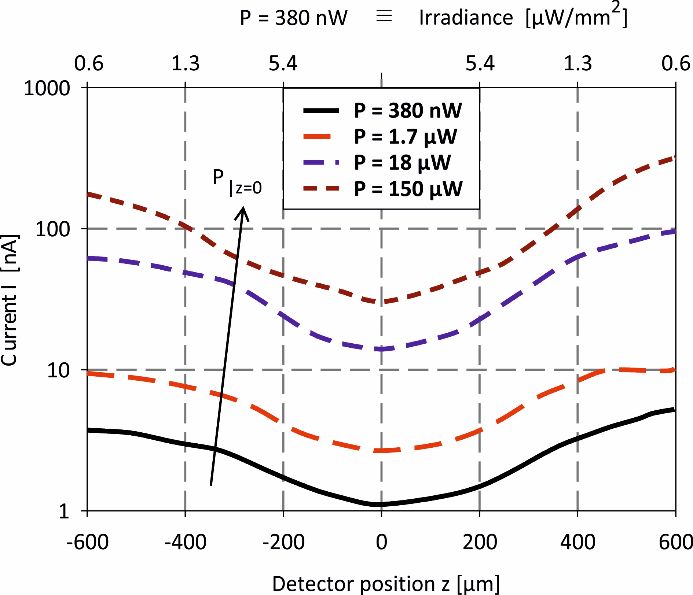


*Figure S3*: *Z-Scan measurement on a-Si:H FIP detectors for different light intensities at 488 nm and 0 V bias. The current breakdown occurs at 380 nW corresponding to intensities < µW/mm² far out of focus.*

To evaluate optimized device operation conditions for optical distance measurements utilizing the focus-induced photoresponse as proposed in the paper, bias dependent z-Scan current measurements have been conducted at 488 nm and at total power of 150 µW. Figure S4 reveals that for 0 V bias, the a-Si:H detector obtains a more pronounced nonlinear current breakdown compared to the z-Scan response at a reverse bias voltage of -1 V. We biased that specific detector type in reverse and forward bias conditions and determined 0 V to be the optimized bias voltage for the distance measurements presented in the paper.


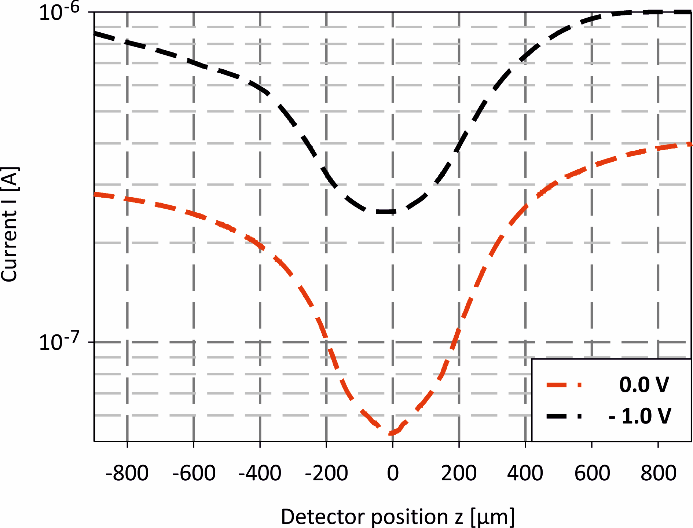


Figure S4: Bias dependent z-Scan current measurement at 488 nm and a total power of 150 µW . For 0 V bias, the a-Si:H detector obtains a more pronounced nonlinear current breakdown compared to a reverse bias voltage.

## **z-Scan simulation irradiances Φ**

Table S 2 shows the photon fluxes, corresponding irradiances and detector positions serving as input parameters for the electro-optical simulations of the z-Scan. Further details on the simulation model, device and material specific parameters for the electro-optical simulations are available on request.

| illumination state | photon flux $\boldsymbol{\Phi}$ $\left[ \frac{\boldsymbol{photons}}{\boldsymbol{cm}^{\boldsymbol{2}}\boldsymbol{\cdot s}} \right]$ | | irradiance $\left[ \frac{\boldsymbol{W}}{\boldsymbol{m}^{\boldsymbol{2}}} \right]$ | detector position in z-Scan technique $\left[ \boldsymbol{mm} \right]$ |
| --- | --- | --- | --- | --- |
| Dark | $\Phi_{0}$ | $0$ | $0$ | $\infty$ |
| Low | $\Phi_{1}$ | ${10}^{14}$ | $0.407$ | $83.4$ |
| moderate | $\Phi_{2}$ | ${10}^{16}$ | $40.7$ | $8.34$ |
| intense | $\Phi_{3}$ | ${10}^{18}$ | $4070.6$ | $0.834$ |

Table S 2: Simulated illumination scenarios at 488 nm including the photon flux as simulation input, corresponding irradiance and the z-displacement of the detector from the focal plane as used in the z‑Scan technique.
